# FasL is more frequently expressed in liver metastases of colorectal cancer than in matched primary carcinomas

**DOI:** 10.1038/sj.bjc.6690202

**Published:** 1999-03

**Authors:** B Mann, A Gratchev, C Böhm, G Demel, B Trojanek, I Schmidt-Wolf, H Stein, C Hanski

**Affiliations:** Department of General Surgery, Universitätsklinikum Benjamin Franklin, Hindenburgdamm 30, Berlin, 12200, Germany; Department of Gastroenterology, Universitätsklinikum Benjamin Franklin, Hindenburgdamm 30, Berlin, 12200, Germany; Department of Pathology, Universitätsklinikum Benjamin Franklin, Hindenburgdamm 30, Berlin, 12200, Germany; Department of Oncology, Universitätsklinikum Rudolf Virchow, Humboldt Universität, Augustenburger Platz 1, Berlin, 13187, Germany

**Keywords:** colorectal carcinoma, progression, metastases, FasL, TILs, immune escape

## Abstract

Colorectal carcinoma cells have recently been shown to express Fas ligand (FasL). This ligand could allow the tumour cells to evade activated tumour-infiltrating lymphocytes (TILs) by inducing their apoptosis and would thus promote tumour survival and possibly metastasis formation. To test this hypothesis in vivo we analysed the expression of FasL mRNA and protein in paired tissue samples of normal colonic mucosa (N), primary colorectal carcinomas (T) and their metastases (M) from a total of 21 patients by four different methods. Additionally, the presence and activation status of infiltrating lymphocytes, which might contribute to the total amount of FasL in the tissue, was determined by semiquantitative reverse transcription–polymerase chain reaction (RT–PCR) in the same samples. The frequency of FasL detection was 30–40% in T and was 60–100% in M, depending on the sensitivity of the method. Simultaneously, the amount of CD25 mRNA, used as a measure of the number of activated TILs, was in 90% of patients lower in M than in T. The increased frequency of FasL detection in liver metastases was therefore not due to the presence of activated TILs. We conclude that metastasizing subpopulations of colorectal tumour cells express FasL more frequently than the primary carcinomas and may be able to eliminate activated TILs in vivo via Fas/FasL-induced apoptosis or other hitherto unknown mechanisms. © 1999 Cancer Research Campaign


*British Journal of Cancer* (1999) **79**(7/8), 1262–1269
© 1999 Cancer Research Campaign
Article no. bjoc.1998.0202

### FasL is more frequently expressed in liver metastases of colorectal cancer than in matched primary carcinomas

**B Mann[1], A Gratchev[2], C Böhm[2], ML Hanski[2], HD Foss[3], G Demel[3], B Trojanek[4], I Schmidt-Wolf[4], H Stein[3], EO Riecken[2], HJ Buhr[1] and C Hanski[2]**

Departments of [1]General Surgery, [2]Gastroenterology and [3]Pathology, Universitätsklinikum Benjamin Franklin, Freie Universität Berlin, Hindenburgdamm 30, 12200 Berlin, Germany and [4]Department of Oncology, Universitätsklinikum Rudolf Virchow, Humboldt Universität Berlin, Augustenburger Platz 1, 13187 Germany

**Summary** Colorectal carcinoma cells have recently been shown to express Fas ligand (FasL). This ligand could allow the tumour cells to evade activated tumour-infiltrating lymphocytes (TILs) by inducing their apoptosis and would thus promote tumour survival and possibly metastasis formation. To test this hypothesis in vivo we analysed the expression of FasL mRNA and protein in paired tissue samples of normal colonic mucosa (N), primary colorectal carcinomas (T) and their metastases (M) from a total of 21 patients by four different methods. Additionally, the presence and activation status of infiltrating lymphocytes, which might contribute to the total amount of FasL in the tissue, was determined by semiquantitative reverse transcription–polymerase chain reaction (RT–PCR) in the same samples. The frequency of FasL detection was 30–40% in T and was 60–100% in M, depending on the sensitivity of the method. Simultaneously, the amount of CD25 mRNA, used as a measure of the number of activated TILs, was in 90% of patients lower in M than in T. The increased frequency of FasL detection in liver metastases was therefore not due to the presence of activated TILs. We conclude that metastasizing subpopulations of colorectal tumour cells express FasL more frequently than the primary carcinomas and may be able to eliminate activated TILs in vivo via Fas/FasL-induced apoptosis or other hitherto unknown mechanisms.

**Keywords:** colorectal carcinoma; progression; metastases; FasL; TILs; immune escape

The Fas molecule (CD95) is constitutively expressed on macrophages and neutrophils and, after activation, also on NK, T and B cells. Most of these cells also express FasL after activation (reviewed in Walker et al, 1997). Cross-linking of Fas with FasL results in apoptosis of these cells, thus preventing their overgrowth and maintaining the immunological homeostasis. Similarly, activated CTLs (cytotoxic T cells) use FasL not only as a cytotoxic effector mechanism to induce apoptosis in Fas-expressing targets (Oshimi et al, 1996), but possibly for an autocrine suicide once the CTLs have eliminated the antigen (Brunner et al, 1995; Dhein et al, 1995). Further, FasL expressed in some peripheral tissues such as testis or the anterior eye chamber induces apoptosis in activated lymphocytes that infiltrate these sites, thus protecting them from immune reaction ('immune privilege') (Bellgrau et al, 1995; Griffith et al, 1995). In several other tissues, such as liver, skin and the colon, FasL may be involved in other cellular processes, e.g. regulation of physiological cell turnover (Galle et al, 1995; French et al, 1996 *a*,*b*; Hahne et al, 1996; Walker et al, 1997).

Colorectal carcinoma (CRC) cells were recently reported to express FasL (O'Connell et al, 1996). Co-culture experiments have shown that these cells as well as FasL$^+$ carcinoma cells of several other tissues are able to induce apoptosis in Jurkat cells (reviewed in Walker et al, 1997). These in vitro experiments, in which Jurkat cells are used as model T cells, are the basis for the hypothesis that, also in vivo, the FasL expression on tumour cells might enable them to evade immune destruction by inducing apoptosis in CTLs (Nagata and Golstein, 1995). The detection of FasL mRNA and protein in the tissue of carcinomas of the lung (Niehans et al, 1997), liver (Strand et al, 1996), skin (Hahne et al, 1996) and colon (Shiraki et al, 1997) supports the concept of a FasL-mediated immune escape of tumour cells.

In vivo model studies have led, however, to somewhat diverging conclusions. In a mouse model, FasL$^+$ melanoma cells rapidly formed tumours in Fas$^+$ mice, whereas tumour growth was delayed in Fas$^-$ *lpr*$^-$ mutant mice, in which immune effectors cannot be killed by FasL (Hahne et al, 1996). On the other hand, gene transfer of FasL into tumour cells was shown to inhibit tumour growth in a mouse model by two mechanisms: either by inducing apoptosis in Fas$^+$ carcinomas or by inducing potent inflammatory reactions in Fas$^-$ carcinomas (Arai et al, 1997).

If FasL expression indeed confers a selective advantage on colon carcinoma cells, one could hypothesize that the FasL$^+$ tumour cell variants are overrepresented in metastatic tissue as a result of successful immune escape during migration and colonization of the distant organs. This notion is supported by the recent investigation of four liver metastases in which the detection of FasL by RT-PCR was more frequent (4/4) than in a group of six primary CRCs (2/6) (Shiraki et al, 1997).

The analysis of the situation in vivo, however, is complicated by the fact that FasL may be expressed on tumour cells as well as on activated T cells. In the normal colonic mucosa, about 10% of T cells are activated and this fraction increases in primary CRC

*Received 18 March 1998*
*Revised 3 July 1998*
*Accepted 14 July 1998*

*Correspondence to:* C Hanski and metastases to 15–25% (Shimizu et al, 1992; Østenstad et al, 1994; De Maria et al, 1996). The activated TILs are likely to express FasL and, unless they are selectively eliminated or FasL$^-$, they might increase the frequency of FasL detection in the primary or metastatic carcinomatous tissue. In the present work, these caveats were taken into account and the validity of the 'counterattack model' was investigated in more detail in vivo. FasL mRNA and protein expression was analysed in paired tissue samples from normal colonic mucosa, primary CRCs and the metastases from a total of 21 patients. Additionally, we examined the presence and activation status of TILs in the same tissue samples. All applied methods led to more frequent detection of FasL in metastases of CRC than in their primary carcinomas. Simultaneously, the number of activated TILs in the metastases was lower than in the primary tumours. The increased frequency of detection of FasL and the decreased number of activated TILs in metastases support the 'counterattack model' in vivo.

### MATERIALS AND METHODS

### Cell lines

Human colonic adenocarcinoma cell lines HT29, LS174T, CaCo2, Colo 201, Colo 205, Colo 206, SW 403, SW 480, SW 620 and T84 were obtained from the American Type Culture Collection. Cell lines HCA7, LS 1034, JWI and CCO7 were kindly provided by W Bodmer, International Cancer Research Fund, London, UK. Troja 1 and Troja 2 cell lines were derived from hepatic metastases of CRC as described previously (Kemmner et al, 1987). All cell lines were cultured under standard conditions.

### Patient tissue samples

Tissue samples from primary colorectal carcinomas, their liver metastases and specimens of normal colonic mucosa (taken at a distance of 10 cm from the tumour) were obtained at the time of surgery from 13 patients with UICC stage IV CRC. Total RNA was immediately isolated from samples of ten patients and protein lysates from samples from six patients. Tissue samples from normal liver was obtained from four patients. Samples of normal, tumour and metastatic tissues from 12 patients (all UICC stage IV) were fixed in 3.7% formalin and embedded in paraffin.

### RNA isolation

Total RNA was extracted from the tissue samples, without previous freezing or storage, with guanidinium thiocyanate followed by centrifugation in caesium chloride as described by Sambrook et al (1989) and from cell lines with RNAzol (Biotex Laboratories, Houston, TX, USA) according to the recommendations of the manufacturer.

### cDNA synthesis

DNase digestion of total RNA was performed prior to cDNA synthesis to rule out contamination with genomic DNA. Twenty micrograms of total RNA was incubated for 2 h in 37°C with 200 U RNase-free DNase I (Boehringer, Mannheim, Germany) diluted in DEPC-water to a final volume of 50 µl containing 50 mM Tris-HCl (pH 6.5), 10 mM magnesium chloride, 10 mM dithiothreitol (DTT) (Gibco BRL, Karlsruhe, Germany) and 20 U of RNase inhibitor (Boehringer, Mannheim, Germany). After denaturation, 2.5 µg of DNase-digested RNA was hybridized with 2.5 µl of random hexanucleotide primers (Gibco BRL, Karlsruhe, Germany) and diluted to a final volume of 22 µl in DEPC-water in 70°C for 10 min. After hybridization, the sample was cooled on ice and 28 µl of mastermix was added, containing 10 µl of 5 × transcriptase buffer (Gibco), 10 µl of dinucleotide triphosphate (dNTP) mix (2.5 mM each), 5 µl of 0.1 mM DTT, 20 U of RNase inhibitor and 500 U of reverse transcriptase (Superscript, Gibco), resulting in a final volume of 50 µl followed by a 1-h incubation at 37°C. The reaction was terminated by boiling for 10 min.

### Primers

The purity and quantity of cDNA were determined in RT-PCR with primers detecting pyruvate dehydrogenase (PDH). PDH forward primer, 5′-GGT ATG GAT GAG GAG CTG GA-3′, is located in exon 1, PDH reverse primer, 5′-CTT CCA CAG CCC TCG ACT AA-3′, is located in exon 2. Consequently, a cDNA amplimer of 103 bp was detected, whereas amplification of genomic DNA contamination resulted in a PCR product of 185 bp. For Fas detection, forward primer, 5′-GAA ATG AAA TCC AAA GCT-3′ (bp 1177–1196), and reverse primer, 5′-TAA TTT AGA GGC AAA GTG GC-3′ (bp 1645–1626), were used, resulting in a 469-bp amplimer. FasL forward primer was 5′-GGA TTG GGC CTG GGG ATG TTT CA-3′ (bp 474–496) and FasL reverse primer 5′-TTG TGG CTC AGG GGC AGG TTG TTG-3′ (bp 817–794), giving a PCR product of 344 bp length. As a marker for the presence of lymphocytes in the tissue samples, CD3 was detected in RT-PCR using CD3 forward primer, 5′-GGC TGT CCT CAT CCT GGC TAT CAT-3′ (bp 21–44), and CD3 reverse primer, 5′-ACT GGT TTC CTT GAA GGT GGC TGT-3′ (bp 535–512), resulting in a specific amplimer of 515 bp. The activation status of resting T-cells was analysed by detecting CD25 with forward primer 5′ TTC CAG GTG AAG AGA AGC CTC AGG 3′ (bp 521–544) and reverse primer 5′ TCT GTT CCC GGC TTC TTA CCA AGA 3′ (bp 807–784) and giving an amplimer of 287 bp length. Primer pairs for Fas and CD25 were created and tested for specificity in the HUSAR® program; primer pairs for FasL and CD3 have been described previously (O'Conell et al, 1996; James-Yarish et al, 1994). All primers were obtained from TIB MOLBIOL Syntheselabor, Berlin, Germany.

### RT-PCR

For every RT-PCR, 20 µg total RNA was reverse transcribed in 50 µl volume as described. Two per cent of the newly synthesized cDNA (1 µl) was added to a 24-µl reaction mixture containing 2.5 µl of a 10 × reaction buffer [100 mM Tris-HCl (pH 8.8), 500 mM potassium chloride and 0.8% Nonidet P40] and 15 mM magnesium chloride (MBI Fermentas, Vilnius, Lithuania), 0.75 µl of a dNTP mixture containing 10 mM dNTP, 10 pmol of each primer and 0.5 U of *Taq* polymerase (MBI, Fermentas). The mixture was overlaid with 50 µl of mineral oil to prevent evaporation. PCR was initiated in a thermal cycler (Omnigene, Hybaid, MWG-Biotech, Germany) and programmed for the following cycle parameters: one cycle of 95°C for 2 min; 35 cycles of 95°C for 45s, 58°C for 45s and 72°C for 1 min; and one cycle of 72°C for 5 min. Fas PCR was performed by adding 2 µl of cDNA to a 23-µl reaction mixture as above apart from a differing magnesium chloride

© Cancer Research Campaign 1999

concentration of 9 mM. The PCR programme for Fas was 95°C, 1 min, 60°C, 1 min, 72°C, 1 min, 40 cycles and 72°C, 5 min, 1 cycle. For FasL PCR, 2.5 µl of template was used in 22.5 µl reaction mixture identical to that of PDH PCR, but with an increased amount of *Taq* polymerase of 1.25 U. Cycle parameters were 95°C, 2 min 1 cycle; 94°C, 45 sec, 65°C, 1 min, 72°C, 1 min, 40 cycles and 72°C, 5 min, 1 cycle. CD3 and CD25 PCR was performed as hot start procedures. One µl of cDNA template was added to 44 µl of reaction mixture containing 5 µl of 10 × reaction buffer (1.5 mM magnesium chloride), 1 µl of dNTP mix and 20 pmol of each primer. The mixture was heated to 95°C for 5 min, allowed to cool to 80°C and then mixed with 1 U of polymerase. RT-PCR parameters for both CD3 and CD25 were 95°C for 1 min, 58°C for 1 min and 72°C for 2 min (40 cycles for CD3 and 30, 35, 40 and 45 cycles for CD25) with one final cycle of 72°C for 5 min. The PCR products were separated by electrophoresis on a 1.5% agarose gel and visualized by ethidium bromide staining. The intensity of the specific bands were estimated as absent (–), weakly positive (±), positive (+) or strongly positive (++).

### In situ hybridization

For in situ hybridization, 5-µm-thick sections of paraffin-embedded tissues were collected on ChemMate™ slides (Dako Diagnostika, Hamburg, Germany). The probe was generated by amplification of cDNA from the anaplastic large-cell lymphoma cell line Karpas 299 (Foss et al, 1997) using primers specific for the FasL sequence. The obtained fragment (820 bp, ranging from base 67 to base 886) was cloned in the vector pTAQ (R&D Systems, Wiesbaden, Germany). The sequence of the probe determined on the DNA sequencer 373 A (Applied Biosystems, Foster City, CA, USA) was identical to the FasL sequence. After linearization of the plasmids, the cRNA probes were obtained by run-off transcription using the T7 RNA polymerase with incorporation of [$^{35}$S]UTP (1300 Ci mmol$^{-1}$, NEN, Bad Homburg, Germany). Alkaline hydrolysis was performed for 50 min to generate cRNA fragments of 100–150 bp in length. For in situ hybridization, dewaxed and rehydrated paraffin sections were exposed to 0.2 N hydrochloric acid and 0.5 mg ml$^{-1}$ Pronase (Boehringer–Mannheim, Germany), acetylated with 0.25% acetic anhydride in 0.1 M triethanolamine pH 8.0 and dehydrated through graded ethanol solutions. Slides were hybridized with 4 × 10$^{5}$ cpm of the labelled probe (specific radioactivity 8 × 10$^{6}$ cpm µg$^{-1}$ RNA). Prehybridization, hybridization, removal of non-specifically bound probe by RNase digestion and washing procedures were performed as described previously (Foss et al, 1997). Autoradiography was performed by dipping the dehydrated slides in Amersham nuclear emulsion (Amersham, Braunschweig, Germany) and exposing for 100 days. The staining of cells was scored by three independent observers for intensity as – (identical to the background), + (2 × stronger than the background) and ++ (more than 2 × stronger than the background).

### Immunohistochemistry

Paraffin-embedded sections (4 µm) were deparaffinized in xylene and rehydrated through graded alcohols into phosphate-buffered saline (PBS). Heat-induced epitope retrieval was achieved by immersion of slides in 10 mM citrate buffer (pH 6.0) and boiling for 10 min in a pressure cooker, followed by cooling in PBS. Endogenous peroxidase was blocked by 15 min incubation with 0.6% hydrogen peroxide in methanol. To prevent unspecific binding, the sections were incubated in 20% inactivated fetal calf serum in PBS for 30 min. They were then incubated in 0.6 µg ml$^{-1}$ mouse monoclonal antibody raised to residues 116–277 of human FasL (Transduction Laboratories, Lexington, KY, USA) overnight at 4°C. After incubation with a second antibody conjugated to peroxidase, the development was performed by incubation in a 3,3′-diaminobenzidine tetrahydrochloride solution. Staining was scored by two independent observers for intensity as – (negative), + (weak), ++ (moderate) and +++ (strong).

### Immunoblotting

Whole-cell lysates from cultured cell lines were prepared from approximately 5 × 10$^{6}$ cells in 10 ml of lysis buffer (1% Triton X-100, 20 mM Tris HCl, 140 mM sodium chloride, 1.5 mM magnesium chloride, 1 mM EGTA, 10% glycerol, 10 µg ml$^{-1}$ DNAase, 1 mM sodium vanadate, 50 mM sodium fluoride, 1 mM PMSF, 1 mM benzamidine and 10 µg ml$^{-1}$ each of aprotinin, leupeptin and pepstatin). Lysates from whole tissue samples were prepared by pulverization of approximately 1 g wet weight of snap-frozen tissue under liquid nitrogen and incubation in 10 ml of lysis buffer. After centrifugation (13 000 × ***g*** for 30 min) and protein determination, 5 µg of total protein was electroblotted onto PVDF membrane following separation on a 12.5% sodium dodecyl sulphate (SDS)-polyacrylamide gel. Membranes were saturated for 1 h using 5% dry milk/1% bovine serum albumin (BSA) in 1 × PBS and incubated overnight at 4°C with 2.5 µg ml$^{-1}$ of anti-human FasL murine monoclonal antibody (Transduction Laboratories) in the same solution. The membranes were then incubated for 1 h with a rabbit anti-murine IgG antibody conjugated to peroxidase (Dako, Hamburg, Germany). The detection followed with the ECL detection system (Amersham, Braunschweig, Germany).

### Statistical evaluation

The *in situ* hybridization data were plotted on scatter diagram and ranked according to the Wilcoxon pair difference test. The differences in gene expression, determined in RT-PCR for T and M were analysed by $\chi^2$-test.

### RESULTS

### Expression of FasL in cell lines derived from colorectal carcinomas and metastases

FasL was detected in several cell lines derived from primary colorectal carcinomas and metastases, at the mRNA level by RT-PCR and at the protein level by immunoblotting, and the correspondence of the two methods was analysed. FasL expression was detectable in 6 of 12 cell lines derived from primary colorectal adenocarcinomas in RT-PCR. Five of these and, additionally, four further cell lines expressed Fas, i.e. 9 of 12 analysed colorectal carcinoma cell lines were Fas$^{+}$. Of four cell lines derived from colorectal carcinoma metastases only one expressed FasL. This cell line, SW620, was Fas$^{-}$, whereas the three other cell lines were Fas$^{+}$ (Figure 1).

The expression of FasL mRNA was further tested on protein level via immunoblotting of lysates from seven cell lines. One of three carcinoma cell lines (HT 29) that were FasL$^{-}$ in RT-PCR

 © Cancer Research Campaign 1999

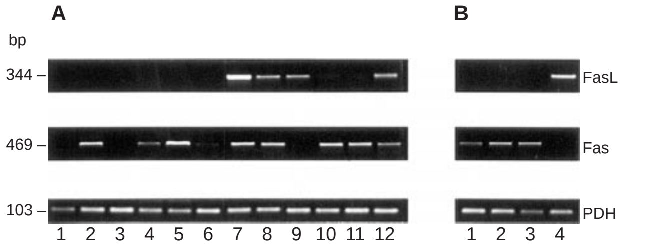

**Figure 1** Detection of Fas- and FasL-mRNA in 12 cell lines derived from human CRC and one melanoma cell line. (**A**) 1, HT29; 2, LS174T; 3, HCA7; 4, LS1034; 5, JWI; 6, Caco2; 7, Colo201; 8, Colo205; 9, Colo206; 10, CCO7; 11, SW403; 12, SW480; and in four cell lines derived from CRC metastases. (**B**) 1, T84; 2, Troja 1; 3, Troja 2; 4, SW620 by RT-PCR. A 344-bp-long FasL-specific sequence or a 469-bp-long Fas-specific sequence was amplified from cDNA, separated on agarose gel and visualized by ethidium bromide staining. RT-PCR of PDH was performed to verify that the amounts of RNA used were similar

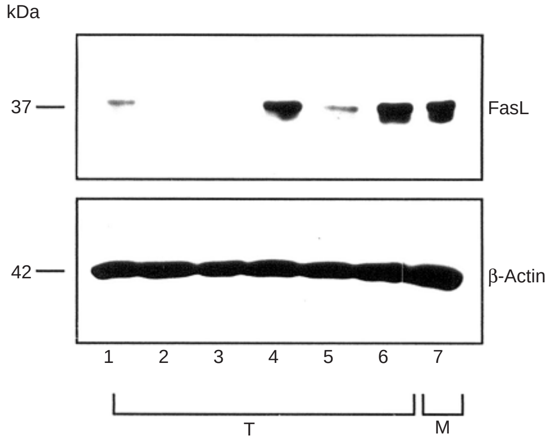

**Figure 2** Immunoblot of 5 μg of protein of whole-cell lysates isolated from six cell lines derived from human primary CRC (T) and one cell line derived from CRC metastases (M) separated on 12.5% gel, blotted and detected with anti-human FasL MAb. 1, HT 29; 2, LS174T; 3, HCA7; 4, CCO7; 5, Colo205; 6, SW480; 7, SW620. Detection with anti-human β-actin MAb was performed to verify that the amounts of protein used were similar showed a weak band at 37 kDa, specific for FasL (Niehans et al, 1997). The second band at $M_r$ 34 500 is assumed to be a differentially glycosylated form of FasL (Suda et al, 1993). All three carcinoma cell lines and the metastatic cell line SW620, which were FasL$^+$ in RT-PCR, showed the specific protein band (Figure 2). These data indicated a satisfactory correspondence between RT-PCR and immunoblotting.

### Expression and localization of FasL-mRNA and -protein in patient tissues

To analyse the expression of FasL mRNA in epithelial cells and lymphocytes in vivo, we performed in situ hybridization with an antisense FasL probe and immunohistochemistry using anti-human FasL murine monoclonal antibody.

### *FasL mRNA as detected by in situ hybridization*

In most samples, FasL mRNA was detectable by in situ hybridization only after 100 or more days of exposure. The usually weak signals after this long exposure time indicated that the expression of FasL mRNA is very low. In the normal colonic mucosa, FasL mRNA expression was detectable only in one case at the tip of the crypt. By contrast, in 4 of 12 primary carcinomas FasL-mRNA expression was detectable in the majority of cells. Of 12 investigated liver metastases six showed expression of FasL mRNA (Figure 3). In 5 of 12 patients all three tissue types showed no staining after 100 days of exposure. In the remaining seven patients, the metastatic tissue stained in one case weaker, in one case equally strong and in five cases stronger than in the corresponding primary tumour. In both primary and metastatic carcinomatous tissues, FasL$^+$ TILs were visible. They were, however, sparse and weakly stained and a difference in their number between primary carcinomas and liver metastases was not evaluated.

In summary, in situ hybridization permitted the detection of FasL mRNA in 30% of primary tumours and in 50% of the metastases. TILs also expressed FasL mRNA in primary tumours and in metastases. These results raised the question of how far activated TILs contribute to the detectable amounts of FasL in patient tissue samples when very sensitive methods such as RT-PCR are used.

### *FasL-protein as detected by immunohistochemistry*

Immunohistochemistry with anti-human FasL monoclonal antibody was performed in sections of tumours and metastases from five patients. The staining of positive cells was diffuse and cytosolic, homogeneously distributed among most epithelial cells (Figure 4). The normal colonic mucosa stained weakly (4/5) or moderately (1/5). The primary tumours were stained moderately (3/5) or strongly (2/5) as were the metastases (2/5 and 3/5 respectively), with no discernible differences between the two groups.

### Expression of FasL-protein as detected by immunoblotting of tissue lysates

In order to quantify FasL protein expression, the lysates from N, T and M of six patients were analysed by immunoblotting. The specific bands for FasL were detectable in two out of six lysates from normal colonic mucosa, in two out of six lysates from primary tumours and in all lysates derived from metastatic tissue (Figure 5). In two patients, all three tissues expressed FasL protein. The intensity of the FasL band was, in one instance in the primary tumour, stronger and in one instance weaker than in the metastatic tissue. In summary, in five out of six investigated patients, the metastatic tissue showed stronger FasL expression than the corresponding primary tumour. Immunoblotting clearly showed that the FasL protein expression in normal tissue is rare. Thus, the weak immunohistochemical staining of the normal mucosa was interpreted to be due to unspecific binding of the antibody to the mucus.

### Expression of FasL-mRNA in normal, carcinomatous and metastatic tissues as detected by RT-PCR

A further refinement of the FasL detection and quantification was achieved through application of semiquantitative RT-PCR. It was applied to RNA isolated from N, T and M tissue of ten patients. Fas mRNA was detected in nine out of ten normal mucosa samples, in nine out of ten primary carcinomas and in all metastases, indicating no significant alteration in the frequency of expression of this gene in colonic tissue during the progression of carcinogenesis. By contrast, FasL mRNA was detectable in five out of ten normal tissues, in three out of ten primary carcinomas

© Cancer Research Campaign 1999

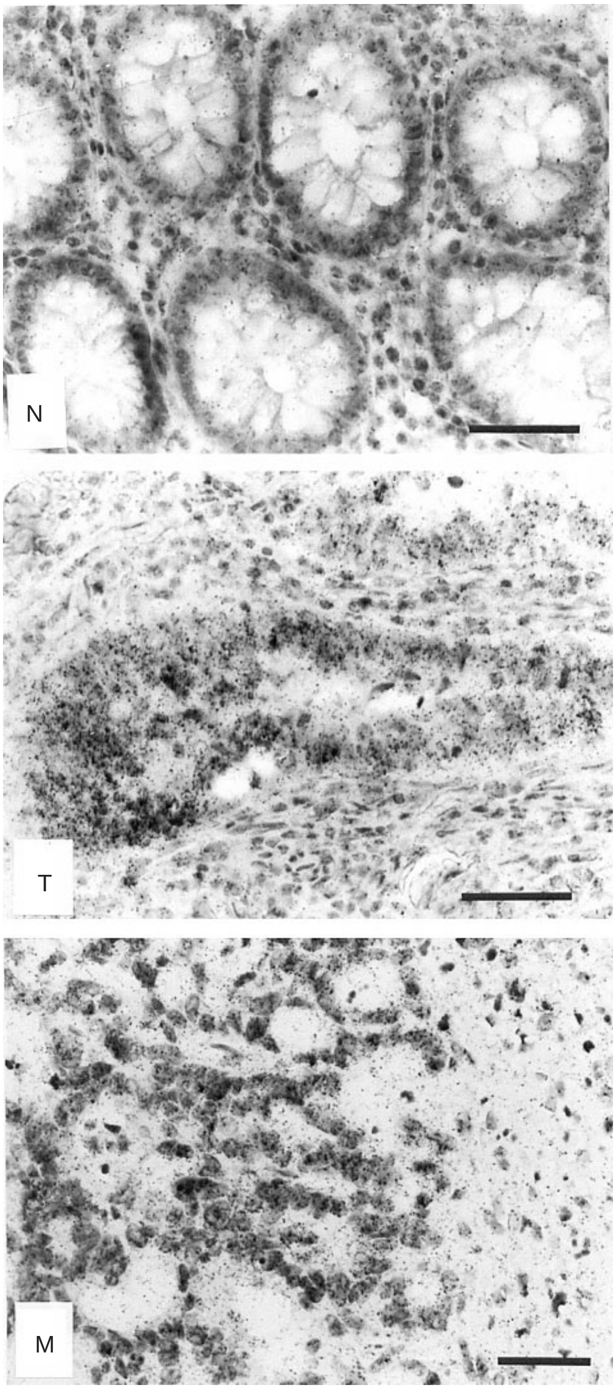

**Figure 3** In situ hybridization of FasL mRNA on paraffin-embedded sections of normal colonic mucosa (N), primary carcinoma (T) and a liver metastasis (M) with FasL antisense probe (see Materials and methods). Counterstaining with haematoxylin–eosin. Bar, 50 µm and in nine out of ten liver metastases. In the four investigated samples of normal liver tissue, Fas mRNA was detected in all and FasL mRNA weakly in three samples (data not shown).

The reverse transcription was carried out with the same amount of RNA from every tissue and the PCR followed with 40 cycles. The semiquantitative evaluation of the specific bands obtained under these standarized conditions indicated that the expression of

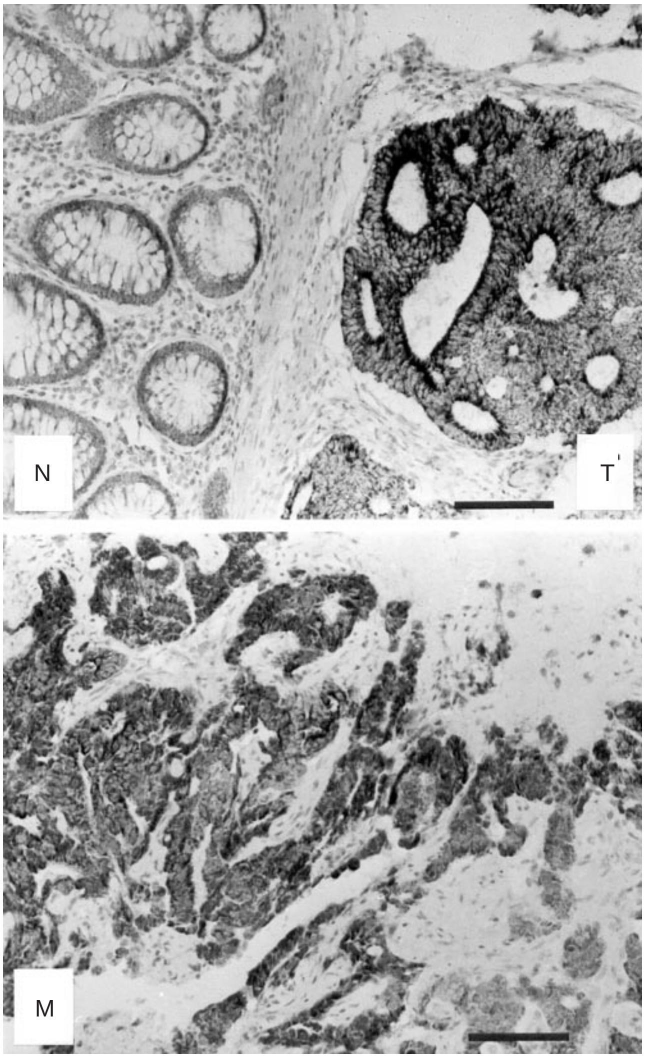

**Figure 4** Immunohistochemistry with anti-human FasL MAb on paraffin-embedded sections of a primary carcinoma (T), the adjacent nondysplastic mucosa (N) and a liver metastasis (M). Bar, 100 µm. A parallel section in which the first antibody was omitted showed no staining. A section of the anterior eye chamber was used as a positive control (data not shown)

FasL was stronger in the metastases than in the primary tumours in nine out of ten cases (Table 1 and Figure 6). Thus, FasL gene expression was significantly more frequent ($P < 0.01$) in the metastatic than in the primary carcinomatous colonic tissue.

### Comparison of FasL mRNA and -protein detection by four methods

The results obtained by the four methods are summarized in Table 2. They indicate that in the normal colonic tissue FasL mRNA is expressed at a very low level and, if the most sensitive method (RT-PCR) is applied, it is detectable in 50% of the samples. In tumour tissue, expression occurs in about 30–40% of cases. In matched liver metastases, FasL was detected most frequently by all methods. The highest frequency of detection was associated with increased amounts of FasL mRNA (Figure 6 and Table 1) and protein (Figure 5) in the metastatic tissue.

 © Cancer Research Campaign 1999

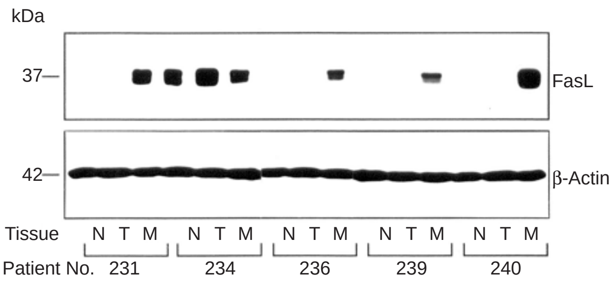

**Figure 5** Immunoblot of 5 μg of protein of lysates obtained from 1 g of normal colonic mucosa (N), primary tumours (T) and metastases (M) from five patients, separated on 12.5% gel, blotted and detected with α-human FasL MAb. Detection with anti-human β-actin MAb was performed to verify that the amounts of protein used were similar

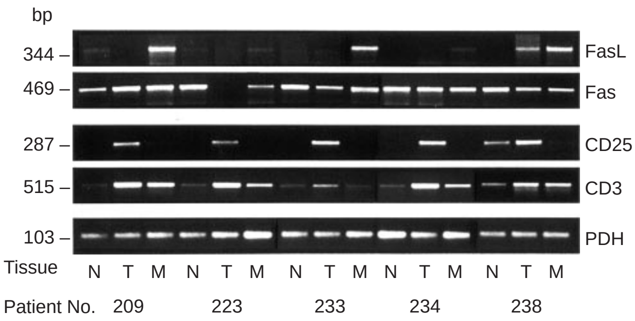

**Figure 6** Detection of Fas, FasL, CD3 and CD25 mRNA in tissue samples from normal colonic mucosa (N), primary carcinomas (T) and their liver metastases (M) by RT-PCR. Specific sequences for each gene were amplified from cDNA, separated on agarose gel and visualized by ethidium bromide staining. RT-PCR of CD3-mRNA was used to detect T cells in the tissue samples; RT-PCR of CD25 mRNA was used to analyse their activation status. RT-PCR of PDH was performed to verify that the amounts of RNA used were similar. Results from five patients out of ten investigated (Table 1) are shown

### Presence and activation status of infiltrating lymphocytes in patients' tissues

The more frequent detection of FasL mRNA and protein in the metastatic tissue prompted the question of how far the detected FasL expression is due to the presence of activated FasL$^+$ TILs.

CD3 mRNA, used as a marker of T cells, was detectable by RT-PCR in nine out of ten samples of N and in all samples of T and M (Figure 6) confirming the previous data on the presence of T cells in these tissues (Shimizu et al, 1992; Østenstad et al, 1994; De Maria et al, 1996). The mRNA of the T-cell activation marker CD25 was detected in three out of ten N samples, in all T samples and in five out of ten metastases after the standard RT-PCR procedure including 40 cycles of PCR. The difference in the frequency of CD25 detection between T and M was significant ($P < 0.01$). In order to analyse further how far this difference is attributable to the different amounts of CD25 mRNA present in total mRNA from the different tissue groups, we performed a RT-PCR of CD25 mRNA in all samples with 30, 35, 40 and 45 cycles, keeping all other parameters constant. The percentage of samples in which CD25 mRNA was detectable increased with increased number of cycles in PCR, and at 45 cycles it reached 80–100% in all samples (Figure 7).

These data indicated that activated T cells are present in all investigated tissues. They suggest, further, that the amount of CD25 mRNA was higher in the primary tumours than in the normal colonic mucosa or metastases. This conclusion is supported by (1) the stronger intensity of the CD25 amplimer bands obtained after 40 PCR cycles from primary tumours than from the paired metastases (Table 1 and Figure 6) and (2) the more frequent detection of CD25 mRNA in primary tumours than in metastases after the same number of cycles (Figure 7). A higher percentage of activated T cells in carcinomatous tissues than in normal colonic mucosa was also detected previously by FACS analysis (Østenstad et al, 1994; De Maria et al, 1996). From these data, we conclude that in 50% of patients the number of activated lymphocytes present in the metastatic tissue was lower than in the primary tumour of similar weight. The increased frequency of detection of FasL in the same metastatic tissues is not, therefore, attributable to the increased number of activated T cells.

### DISCUSSION

The 'counterattack model' suggesting that tumour cells can induce apoptosis of cytotoxic T cells via Fas/FasL death signal, is based on in vitro data, which have shown that different FasL$^+$ tumour cells are able to kill Jurkat cells in co-culture experiments. It was

**Table 1** Detection of FasL and CD25 mRNA by means of RT-PCR in paired samples of normal colonic mucosa (N), primary carcinomas (T) and liver metastases (M) from ten patients. RT-PCR was carried out for 40 cycles. The intensity of the amplimer bands was semiquantitatively estimated after ethidium bromide staining. The change in the intensity of amplimer bands for each gene was additionally evaluated within the paired M and T samples (M vs. T) and the extent of increase (↑) or decrease (↓) was estimated (nc, not changed). The PDH band had a similar intensity (++) in all samples. The representative electrophoresis results are shown in Figure 6

| | N | | T | | M | | M vs. T | |
|---|---|---|---|---|---|---|---|---|
| **Patient no.** | **FasL** | **CD25** | **FasL** | **CD25** | **FasL** | **CD25** | **FasL** | **CD25** |
| 201 | – | ± | – | ++ | + | – | ↑ | ↓↓ |
| 209 | ± | – | – | ++ | ++ | ± | ↑↑ | ↓ |
| 212 | + | – | – | ++ | + | + | ↑ | ↓ |
| 214 | + | + | + | + | ++ | + | ↑ | nc |
| 223 | ± | – | – | ++ | ± | – | ↑ | ↓↓ |
| 229 | + | – | – | ++ | ++ | – | ↑↑ | ↓↓ |
| 232 | – | – | – | ++ | – | + | nc | ↓ |
| 233 | – | – | ± | ++ | ++ | – | ↑ | ↓↓ |
| 234 | – | – | – | ++ | ± | – | ↑ | ↓↓ |
| 238 | – | + | + | ++ | ++ | ± | ↑ | ↓ |
| Total detectable | 5/10 | 3/10 | 3/10 | 10/10 | 9/10 | 5/10 | 9/10 ↑ | 9/10↓ |

© Cancer Research Campaign 1999

**Table 2** Frequency of FasL+ samples in normal colonic mucosa (N), primary carcinomas (T) and liver metastases (M) determined by four different methods. Only strongly stained samples (+++) in the immunohistochemistry group were included

| Detection method | N | T | M |
|---|---|---|---|
| Immunohistochemistry | 0/5 | 2/5 | 3/5 |
| Immunoblotting | 2/6 | 2/6 | 6/6 |
| In situ hybridization | 1/12 | 4/12 | 6/12 |
| RT-PCR | 5/10 | 3/10 | 9/10 |

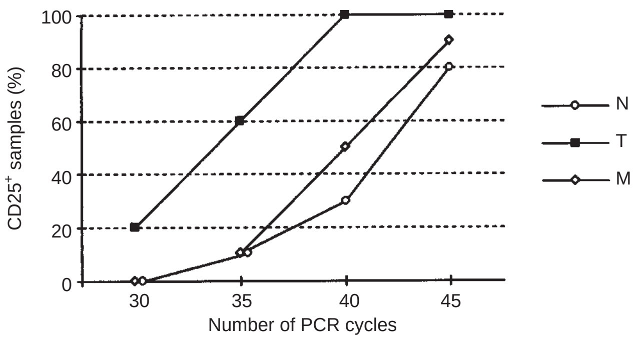

**Figure 7** Percentage of CD25+ samples determined in RT-PCR after 30, 35, 40 and 45 cycles in normal colonic tissue (N), primary tumours (T) and liver metastases (M) (each *n* = 10).

hypothesized that an analogous mechanism facilitating the progression of the carcinomas is operative in vivo. The main goal of the present work was to test this hypothesis by analysing the expression of FasL mRNA and protein during the progression of CRC.

We detected Fas mRNA in 9 out of 12 cell lines derived from primary CRC and in three out of four metastatic cell lines. Thus, a considerable percentage of tumour- and metastasis-derived cell lines should be susceptible to FasL-mediated apoptosis. In patient tissues, Fas mRNA was detected in almost all samples in all three tissue types. Previous immunhistochemical data have shown, however, that colon carcinoma tissue expresses less Fas than normal colonic mucosa. This down-regulation may protect some tumour cells from killing by apoptosis (Möller et al, 1994).

FasL was detected in 6 out of 12 investigated cell lines derived from primary colorectal carcinomas. A similar result was obtained by other authors, who detected FasL in about 50% of investigated colon carcinoma cell lines (O'Connell et al, 1996; Arai et al, 1997; Shiraki et al, 1997). The RT-PCR data at the protein level were confirmed by immunoblotting of whole-cell lysates. The low percentage of FasL+ cell lines derived from metastases (one of four) detected in the present work was surprising and did not support the hypothesis of preferential expression of FasL in metastatic cells; the number of available cell lines is, however, too low to yield statistically significant data. It is of interest that only the FasL+ metastatic cell line SW620 expressed no detectable Fas, i.e. it was resistant to FasL-mediated apoptosis.

In vivo, FasL was detectable in about 10–30% of the normal mucosa samples in immunoblots, by immunhistochemistry and in situ hybridization. Using RT-PCR, a more sensitive method, we found FasL mRNA in 50% of samples of normal mucosa, in 30% of the primary tumours and in 90% of the corresponding metastases. With the four methods used for analyses of FasL mRNA and protein in patient tissues, the percentage of FasL+ samples varied in primary tumours between 30% and 40% and in metastases between 50% and 100%. However, all procedures yielded a higher frequency of FasL detection in the metastases than in the primary tumours. This difference was significant when RT-PCR, the most sensitive method, was used (Table 2). Thus, the situation in cell lines derived from metastases does not reflect the findings found in metastatic tissue samples by us and others. This might be due to the complex cytokine environment and the presence of co-stimulatory factors, which may induce FasL expression in vivo but are missing in cell cultures, or to other FasL+ cells in the tissue.

The question of how far the activated TILs contribute to the detected amounts of FasL was addressed by analysing the expression of CD3 and CD25 in all samples. CD3 mRNA was detectable in almost all specimens of the normal colon and in all carcinomas and metastases, as expected from previous FACS data (Shimizu et al, 1992; Østenstad et al, 1994; De Maria et al, 1996). The amount of CD25 mRNA marking the activated subpopulation of T cells was considerably higher in the primary carcinomas than in the normal colonic mucosa (Table 1 and Figures 6 and 7). This finding corroborates reports that show in FACS analysis a higher percentage of activated T cells in primary colorectal carcinomas than in normal mucosa (Østenstad et al, 1994; De Maria et al, 1996). In liver metastases, the amount of CD25 mRNA was in nine out of ten samples lower than in the paired primary carcinomas and was similar to the amount in normal tissue. Assuming that the CD25 amounts correctly reflect the number of activated TILs in the three tissues, these data suggest that the number of activated TILs in M is frequently lower than in T; they either infiltrate the metastases less efficiently than the primary tumours or are eliminated in the metastatic tissue by FasL-mediated apoptosis or other mechanisms. The concomitant, more frequent, FasL expression in the metastases suggests that the detection of FasL mRNA in the metastatic tissue samples indeed represents expression on the metastatic tumour cells and is not due to increased presence of activated TILs.

In FasL+ tumours, there seems to be a potentially lethal reciprocal interaction; both tumour cells and activated TILs are expressing Fas and FasL and, therefore, both have the ability to act either as effectors or as targets in Fas/FasL-mediated killing. Thus, the survival of the tumour or the host cells may depend on which cells can accomplish which task more efficiently. Fas+ tumours are potentially susceptible to FasL-mediated killing. It has been shown in vitro, however, that Fas+ tumour cells may fail to respond to FasL signal with apoptosis (O'Connell et al, 1996). More importantly, in tumour or metastatic tissue, tumour cells greatly outnumber the TILs. This disproportion increases the probability that tumour cells may kill the cytotoxic T lymphocytes rather than the reverse, as shown recently by Zeytun et al (1997) for lymphoma cells in vitro. This hypothesis of deletion of tumour-reactive T cells via Fas–FasL interaction could explain why the amount of CD25 antigen found in the metastases was lower than in the tumours.

This notion is further supported by the results of Hahne et al (1996), who observed that FasL+ tumours grow faster in Fas+ than in Fas− mice. In other model systems, however, FasL expression on tumour cells, including colorectal carcinomas, does not facilitate tumour growth but induces tumour rejection, mediated mainly by neutrophils (Arai et al, 1997; Seino et al, 1997). When FasL+ pancreatic islet cells were injected into allogeneic hosts, they did not induce apoptosis of the infiltrating T cells but were destroyed

© Cancer Research Campaign 1999

by neutrophilic granulocytes (Kang et al, 1997). Furthermore, T cells activated by viral infection are not susceptible to FasL-mediated cell death in vivo (Ehl et al, 1996). Taken together, these results suggest that FasL-expressing cells can prevent immune response by inducing cell death of activated lymphocytes, and this may be the case in colon carcinoma metastases. Alternatively, FasL$^+$ cells may induce potent granulocytic inflammatory response, and it was hypothesized recently that this induction is mainly attributable to soluble FasL (Strasser and O'Connor, 1998). The factors necessary for this reaction are apparently not present in sufficient amounts in the tumour and metastatic tissue because acute granulocytic inflammation is only rarely observed in these tissues. This may be due to a number of mechanisms that suppress the lymphocyte activation in tumour tissues, such as low number of dendritic cells in the tumour (Ambe et al, 1989), reduced IL-4 and TNF-$\alpha$ expression (Barth et al, 1996) or the loss of the $\zeta$-chain of the T-cell receptor and granzyme B-mRNA in TILs of human CRC (Mulder et al, 1997). These defects in TILs may additionally enhance their susceptibility to FasL-mediated apoptosis and suppress the inflammatory reaction.

In summary, we showed by four different methods that the frequency of FasL mRNA and protein expression in liver metastases of human colorectal cancers is higher than in their primary carcinomas. Simultaneously, the number of activated TILs is decreased in these metastatic tissues. We conclude that FasL expression could give metastasizing subpopulations of CRC a growth advantage and might facilitate metastatic growth in distant organs. This might be attributable to elimination of activated TILs via Fas/FasL induced apoptosis or to other hitherto unknown mechanisms.

### ACKNOWLEDGEMENT

This work was supported by DFG, Proj. No. Ma-1989 1/1, Deutsche Krebshilfe 10-1025-HaI and Sonnenfeld Stiftung.

### REFERENCES

Ambe K, Mori M and Enjoji M (1989) S-100 protein positive dendritic cells in colorectal adenocarcinomas. *Cancer* **63**: 496–503

Arai H, Gordon D, Nabel EG and Nabel GJ (1997) Gene transfer of Fas ligand induces tumour regression *in vivo*. *Proc Natl Acad Sci USA* **94**: 13862–13867

Barth RJ, Camp BJ, Martuscello TA, Dain BJ and Memoli VA (1996) The cytokine microenvironment of human colon carcinoma. *Cancer* **78**: 1168–1178

Bellgrau D, Gold D, Selawry H, Moore J, Franzusoff A and Duke RC (1995) A role of CD95 ligand in preventing graft rejection. *Nature* **377**: 630–632

Brunner TH, Mogli RJ, LaFace D, Yoo NJ, Mahboubi A, Echeverri F, Martin SJ, Force WR, Lynch DH, Ware CF and Green DR (1995) Cell autonomous Fas/Fas-ligand interaction mediates activation-induced apoptosis in T-cell hybridomas. *Nature* **373**: 441–444

De Maria R, Boirivant M, Cifone MG, Roncaioli P, Hahne M, Tschopp J, Pallone F, Santoni A and Testi R (1996) Functional expression of Fas and Fas Ligand on human gut lamina propria T lymphocytes. *J Clin Invest* **97**: 316–322

Dhein J, Walczak H, Bäumler C, Debatin KM and Krammer PH (1995) Autocrine T-cell suicide mediated by APO-1/(Fas/CD95). *Nature* **373**: 438–441

Ehl S, Hoffmann-Rohrer U, Nagata S, Hengartner H and Zinkernagel R (1996) Different susceptibility of cytotoxic T-cells to CD95 ligand-mediated cell death after activation *in vitro* vs *in vivo*. *J Immunol* **156**: 2357–2360

Foss HD, Demel G, Anagnostopoulos I, Aranjo I, Hummel M and Stein H (1997) Uniform expression of cytotoxic molecules in anaplastic large cell lymphoma of Null/T-cell phenotype and in cell lines derived from anaplastic large cell lymphomas. *Pathobiology* **65**: 83–90

French LE and Tschopp J (1996) Constitutive Fas Ligand expression in several non-lymphoid mouse tissues and implications for immune-protection and cell turnover. *Behring Inst Mitt* **97**: 156–160

French LE, Hahne M, Vicard I, Radlgruber G, Zanone R, Becker K, Möller C and Tschopp J (1996) Fas and Fas ligand in embryos and adult mice: ligand expression in several immune privileged tissues and coexpression in adult tissues characterized by apoptotic cell turnover. *J Cell Biol* **133**: 335–343

Galle PR, Hofmann WJ, Walczak H, Schaller H, Otto G, Stremmel W, Krammer PH and Runkel L (1995) Involvement of the CD95 receptor and ligand in liver damage. *J Exp Med* **182**: 1223–1230

Griffith TS, Brunner TH, Fletcher SM, Green DR and Ferguson TA (1995) Fas ligand induced apoptosis as a mechanism of immune privilege. *Science* **270**: 1189–1192

Hahne M, Rimoldi D, Schröter M, Romero P, Schreier M, French LE, Schneider P, Bornand T, Fontana A, Lienard D, Cerottini JC and Tschopp J (1996) Melanoma cell expression of Fas(Apo-1/CD95) Ligand: implications for tumour immune escape. *Science* **274**: 1363–1366

James-Yarish M, Bradley WG, Emmanuel PJ, Good RA and Day NK (1994) Detection of cell specific cluster determinant expression by reverse transcription polymerase chain reaction. *J Immunol Meth* **169**: 73–82

Kang SM, Schneider DB, Lin Z, Hanahan D, Dichek DA, Stock PG and Baekkeskov S (1997) Fas ligand expression in islets of Langerhans does not confer immune privilege and instead targets them for rapid destruction. *Nature Med* **3**: 738–743

Kemmner W, Schlag P and Brossmer R (1987) A rapid and simple procedure for dissociation of tumour tissue from the human colon. *J Cancer Res Clin Oncol* **113**: 400–401

Möller P, Koretz K, Leihäuser F, Brüderlein S, Henne C, Quentmeier A and Krammer PH (1994) Expression of APO-1 (CD95), a member of the NGF/TNF receptor superfamily, in normal and neoplastic colon epithelium. *Int J Cancer* **57**: 371–377

Mulder WMC, Bloemena E, Stukart MJ, Kummer JA, Wagstaff RJ and Scheper RJ (1997) T-cell receptor-zetta and granzyme B expression in mononuclear cell infiltrates in normal colonic mucosa and colon carcinoma. *Gut* **40**: 113–119

Nagata S and Golstein P (1995) The Fas death factor. *Science* **267**: 1449–1456

Niehans GA, Brunner T, Frizelle SP, Liston JC, Salerno T, Knapp DJ, Green DR and Kratzke RA (1997) Human lung carcinomas express Fas-ligand. *Cancer Res* **57**: 1007–1012

O'Connell J, Sullivan GC and Collins JK (1996) The Fas counterattack: Fas-mediated T-cell killing by colon cancer cells expressing Fas-Ligand. *J Exp Med* **184**: 1075–1082

Oshimi Y, Oda S, Honda Y, Nagata S and Miyazaki S (1996) Involvement of Fas Ligand and Fas mediated pathway in the cytotoxicity of human natural killer cells. *J Immunol* **157**: 2909–2915

Østenstad B, Lea T, Schlichting E and Harboe M (1994) Human colorectal tumour infiltrating lymphocytes express activation markers and the CD45R0 molecule, showing a primed population of lymphocytes in the tumour area. *Gut* **35**: 382–387

Sambrook J, Fritsch EF and Maniatis T (1989) *Molecular Cloning, a Laboratory Manual*. Cold Spring Harbor Laboratory Press: Cold Spring Harbor, NY

Seino KI, Kayagaki N, Okumura K and Yagita H (1997) Antitumour effect of locally produced CD95 ligand. *Nature Med* **3**: 165–170

Shimizu Y, Watanabe A and Whiteside TL (1992) Memory T-lymphocytes are the main population of tumour infiltrating lymphocytes obtained from human primary liver tumours. *J Hepatol* **16**: 197–202

Shiraki K, Tsuji N, Shioda T, Isselbacher J and Takahashi H (1997) Expression of Fas-ligand in liver metastases of human colonic adenocarcinomas. *Proc Natl Acad Sci USA* **94**: 6420–6425

Strand S, Hofmann WJ, Hug H, Müller M, Otto G, Strand D, Mariani SM, Stremmel W, Krammer PH and Galle PR (1996) Lymphocyte apoptosis induced by CD95 (APO-1/Fas) ligand-expressing tumour cells – a mechanism of immune evasion? *Nature Med* **2**: 1361–1366

Strasser A and O'Connor L (1998) Fas ligand – caught between scylla and charybdis. *Nature Med* **4**: 21–23

Suda T, Takahashi T, Golstein P and Nagata S (1993) Molecular cloning and expression of FasL, a novel member of the tumor necrosis factor family. *Cell* **75**: 1169–1178

Walker PR, Saas P and Dietrich PY (1997) Role of FasL in immune escape – the tumour cell strikes back. *J Immunol* **158**: 4521–4524

Zeytun A, Hassuneh M, Nagarkatti M and Nagarkatti PS (1997) Fas–Fas ligand-based interactions between tumour cells and tumour-specific cytotoxic T lymphocytes: a lethal two-way street. *Blood* **90**: 1952–1959

© Cancer Research Campaign 1999 
